# The role of radiographic knee alignment in total knee replacement outcomes and opportunities for artificial intelligence-driven assessment

**DOI:** 10.1016/j.ocarto.2026.100889

**Published:** 2026-10-16

**Authors:** Zhisen Hu, Dominic Cullen, David S. Johnson, Aleksei Tiulpin, Timothy F. Cootes, Claudia Lindner

**Affiliations:** a Department of Informatics, Imaging and Data Sciences, The University of Manchester, UK; b Northern Care Alliance NHS Foundation Trust, UK; c Department of Trauma and Orthopaedics, Stockport NHS Foundation Trust, Stepping Hill Hospital, UK; d School of Health and Society, University of Salford, UK; e School of Biological Sciences, The University of Manchester, UK; f Weill Cornell Medicine, Cornell University, USA; g Research Unit of Health Sciences and Technology, University of Oulu, Finland

**Keywords:** Artificial intelligence, Knee alignment, Knee replacement, Radiographs

## Abstract

Prevalent knee osteoarthritis (OA) imposes substantial burden on health systems with no cure available. Its ultimate treatment is total knee replacement (TKR). Complications from surgery and recovery are difficult to predict, and numerous factors may affect them. Radiographic knee alignment is a key factor affecting TKR success, affecting outcomes such as postoperative pain or function. Recently, artificial intelligence (AI) has been introduced to automatically analyse knee radiographs, for example, to assess knee alignment. These tools reduce measurement variability, support high-throughput analysis of large imaging datasets, and help identify alignment features associated with postoperative outcomes. A focused review is therefore needed to clarify how knee alignment biomarkers are measured, how they relate to TKR outcomes, and how AI-based approaches may support their assessment and clinical application in surgical planning and patient risk stratification.

In this review, we first examine the current scoring protocols for evaluating TKR outcomes and potential knee alignment biomarkers associated with these outcomes. We then discuss existing AI-based approaches for generating knee alignment biomarkers from knee radiographs, and explore future directions for AI-driven knee alignment assessment and TKR outcome prediction.

Knee alignment biomarkers represent an important but underexplored domain for improving prediction of TKR outcomes. AI-driven radiographic assessment offers a promising means to generate consistent, scalable, and clinically actionable measures of alignment, though challenges remain in accuracy, validation, multi-view integration, and clinical deployment. Improved models, rigorous external validation, and systems that link alignment measurements to outcome prediction are needed to enhance reliability and clinical applicability.

## 1 Introduction

Knee osteoarthritis (OA) is one of the most widespread and burdensome health problems [1], [2], [3], [4]. Total knee replacement (TKR) may be offered as treatment for end-stage knee OA. Nevertheless, TKR is an invasive procedure involving prosthesis implantation at the knee joint, and around 10% of patients are dissatisfied following TKR [5], [6]. Dissatisfaction is often assessed through patient-reported outcome measures (PROMs) [7]. In clinical practice, predicting poor TKR outcomes in advance could help optimise patient selection and improve management strategies.

Radiographic knee alignment is an important biomarker for predicting TKR outcomes and long-term joint health. Abnormalities such as femoral or tibial deformities can directly influence surgical planning, implant selection, and postoperative recovery [8], [9]. Traditional alignment measurement is manual, time-consuming, and requires full-leg radiographs, which are not always undertaken in clinical practice. Instead, standard weight-bearing anteroposterior (AP) knee radiographs are often the main imaging modality. Automated methods for alignment assessment in standard weight-bearing knee radiographs are potentially clinically valuable for improving efficiency in the knee OA treatment pathway.

Machine learning and deep learning have been widely applied in medical image analysis including for knee alignment assessment [10], [11], [12], [13]. However, many current review articles in knee image analysis focus on knee OA diagnosis [14], [15], [16], [17], [18] or MRI-based knee segmentation [19], [20], [21], [22], not considering the assessment of knee alignment in knee radiographs. To address this gap, this article provides a comprehensive review of the current literature on radiographic knee alignment factors influencing TKR outcomes and on AI-based techniques for automated alignment assessment.

This review article aims to: i) Explore possible radiographic knee alignment biomarkers for TKR outcomes and relevant techniques for their assessment. ii) Investigate and summarise the current literature on AI-based techniques used to assess radiographic knee alignment for TKR outcomes. iii) Discuss available commercial products for automated knee alignment measurement. iv) Identify gaps and possible future directions for generating automated knee alignment biomarkers for TKR outcomes and predictions of TKR outcomes.

## 2 Clinical and radiographic determinants of TKR outcomes

### 2.1 Patient-reported outcome measures (PROMs) scoring protocols for TKR

Specific scoring protocols are used to pre- and postoperatively collect PROMs in TKR patients. These protocols are important to TKR outcome evaluation and prediction. They can indicate if a patient’s OA status negatively affects their quality of life in terms of both pain and function. However, these instruments differ in purpose. Some were developed specifically for assessing outcomes after knee arthroplasty, whereas others were originally designed for knee osteoarthritis (OA) assessment and were later applied to TKR cohorts. Therefore, their suitability depends on whether the aim is to assess preoperative OA severity, postoperative recovery, or change in symptoms and function after surgery.

The Knee Society Score [23] is a knee arthroplasty-specific outcome measure that combines clinician-assessed evaluation of pain, range of motion, stability, and alignment with assessment of functional activities such as walking and stair climbing, and it has been widely used to assess patients before and after TKR. The Oxford Knee Score (OKS) [24] is a 12-item patient-reported outcome measure specifically developed to assess pain and function after TKR and is commonly administered both preoperatively and postoperatively to quantify surgical outcome. In contrast, the Knee Injury and Osteoarthritis Outcome Score (KOOS) [25] and the Western Ontario and McMaster Universities Osteoarthritis Index (WOMAC) [26] are broader knee OA instruments. KOOS was developed to assess symptoms, pain, activities of daily living, sport/recreation, and knee-related quality of life in patients with knee injury or OA, and it has also been validated for use in total knee replacement populations. WOMAC was originally developed for patients with hip or knee OA and evaluates pain, stiffness, and physical function; it is therefore particularly suitable for assessing OA-related disease burden before TKR, but it has also been used to evaluate postoperative changes after TKR. An overview of these scoring systems is listed in Table 1.

**Table 1:** Scoring protocols for patient-reported outcome measures regarding knee replacement surgery.

Scoring protocols	Assessment	Grading Scale
KSS	Knee pain, RoM (range of motion), stability, walking function, stair climbing function	Clinical rating system (0–100): knee pain 0-50, RoM: 0–25 points, stability: 0–25. Functional scoring system (0–100): walking: 0–50, stair climbing: 0–50. The higher the better.
OKS	12 questions regarding knee health, pain severity, stiffness, daily activities	Excellent (41–48): minimal symptoms as well as excellent knee function. Good (34–40): some symptoms, but good knee function. Fair (27–33): moderate symptoms, and knee function. Poor (0–26): significant symptoms, and poor knee function.
KOOS	Impact of knee injury and OA on an individual’s quality of life	Pain: 0–100. Symptoms: 0–100. Activities of daily living: 0–100. Sport and recreation function: 0–100. Knee-related quality of life: 0–100. The higher the better.
WOMAC	24 questions regarding function, pain, and stiffness	Pain subscale: 5 questions, 0–20. Stiffness: 2 questions, 0–8. Physical function: 17 questions, 0–68. The lower the better.

### 2.2 Alignment biomarkers for TKR outcomes

Knee alignment is a key radiographic biomarker for predicting TKR outcomes, influencing surgical planning, implant selection, and postoperative results. In this section, we introduce several knee alignment biomarkers relevant to TKR outcomes. To distinguish these different clinical roles, the alignment biomarkers summarised in Table 2 are organised into preoperative and postoperative predictors.

**Table 2:** Knee alignment biomarkers associated with TKR outcomes.

Studies	Pre-/Post-operative Predictive Markers	Biomarkers	Conclusions	Number of Participants
[27]	Pre-operative	Preoperative varus deformities	More varus deformities lead to higher improvement rates measured by KOOS	110 patients, 19 males and 91 females
[28]		Preoperative OA progression, joint angle	More severe knee OA and higher joint angles were associated with better (lower) postoperative WOMAC scores	172 patients, 70 males and 102 females
[9]		Preoperative varus/valgus alignment	Excessive alignment has a greater risk of failure	5342 TKRs (3699 patients), 1457 males and 2242 females
[8]	Post-operative	Postoperative tibiofemoral alignment	The neutrality of alignment maximize the implant survival	6070 TKRs (3992 patients), 1556 males and 2436 females
[29]		Postoperative coronal alignment	Higher incidence of revision surgery	6070 TKRs (3992 patients), 1556 males and 2436 females
[30]		Postoperative coronal TKR alignment	Neutral limb alignment and higher KSS are associated only in patients with preoperative non-varus alignment	38 patients, 16 males and 22 females
[31]		Postoperative alignment	Postoperative malalignment is a risk factor for long-term component failure	280 patients, 142 males and 138 females
[32]		Postoperative prosthetic alignment	Neither mechanical axis, nor component alignment, is associated with dissatisfaction (OKS) at one year following TKR	211 patients, 230 TKRs, 105 males and 106 females

Severe preoperative varus deformity has been linked to greater KOOS improvements following TKR [27]. Similarly, more advanced OA progression and larger preoperative joint angles have been associated with better postoperative WOMAC outcomes [28]. However, preoperative varus and valgus malalignment have also been linked to an increased risk of revision surgery [9]. These findings suggest that while severe deformity may permit greater functional improvement after surgery because of a lower preoperative baseline, excessive malalignment may also increase the long-term risk of revision.

Postoperative alignment has also been associated with TKR outcomes. Residual varus or valgus malalignment after surgery has been linked to a higher incidence of revision surgery [8], [29] and an increased risk of long-term component failure [31]. Neutral postoperative limb alignment has been associated with higher KSS, although this relationship was observed only in patients without preoperative varus deformity [30]. However, not all studies have demonstrated a clear association between postoperative alignment and patient-reported outcomes. For example, Huijbregts et al. [32] found that neither mechanical axis nor component alignment was associated with one-year postoperative satisfaction measured using OKS.

Tibiofemoral joint deformities could be measured by angles including anatomical femorotibial angle (FTA) (shown in Fig. 1a), the hip-knee-ankle angle (HKAA) (shown in Fig. 1b), medial proximal tibial angle (shown in Fig. 1c), and lateral distal femoral angle (shown in Fig. 1c). FTA and HKAA may also be calculated using their supplementary angles [11], [12], [13], [33]. Angles are generally measured relative to anatomical or mechanical axes. For lower limbs, the mechanical axis is commonly measured between the center of the femoral head and the center of the knee (for the femur) and from the center of the knee to the ankle (for the tibia), whereas the anatomical axis is a line drawn proximal to distal in the intramedullary canal bisecting the femur and tibia in one-half. Anatomical FTA represents the angle formed by the intersection of the femoral and tibial anatomical axes at the knee, with deviations from the normal angle indicating misalignment or pathology [34]. Medial proximal tibial angle measures the angle between the tibial mechanical axis and the proximal tibial joint line, aiding knee deformity evaluation and surgical planning [35]. Lateral distal femoral angle is the angle between the femoral mechanical axis and the distal femoral joint line on the lateral side, serving a similar role to the above-mentioned angles [35]. HKAA is defined as the angle between the femoral and tibial mechanical axes [36] and can measure coronal plane knee alignment even better than anatomical FTA, as it includes load distribution within the knee joints [33].

**Fig. 1:**
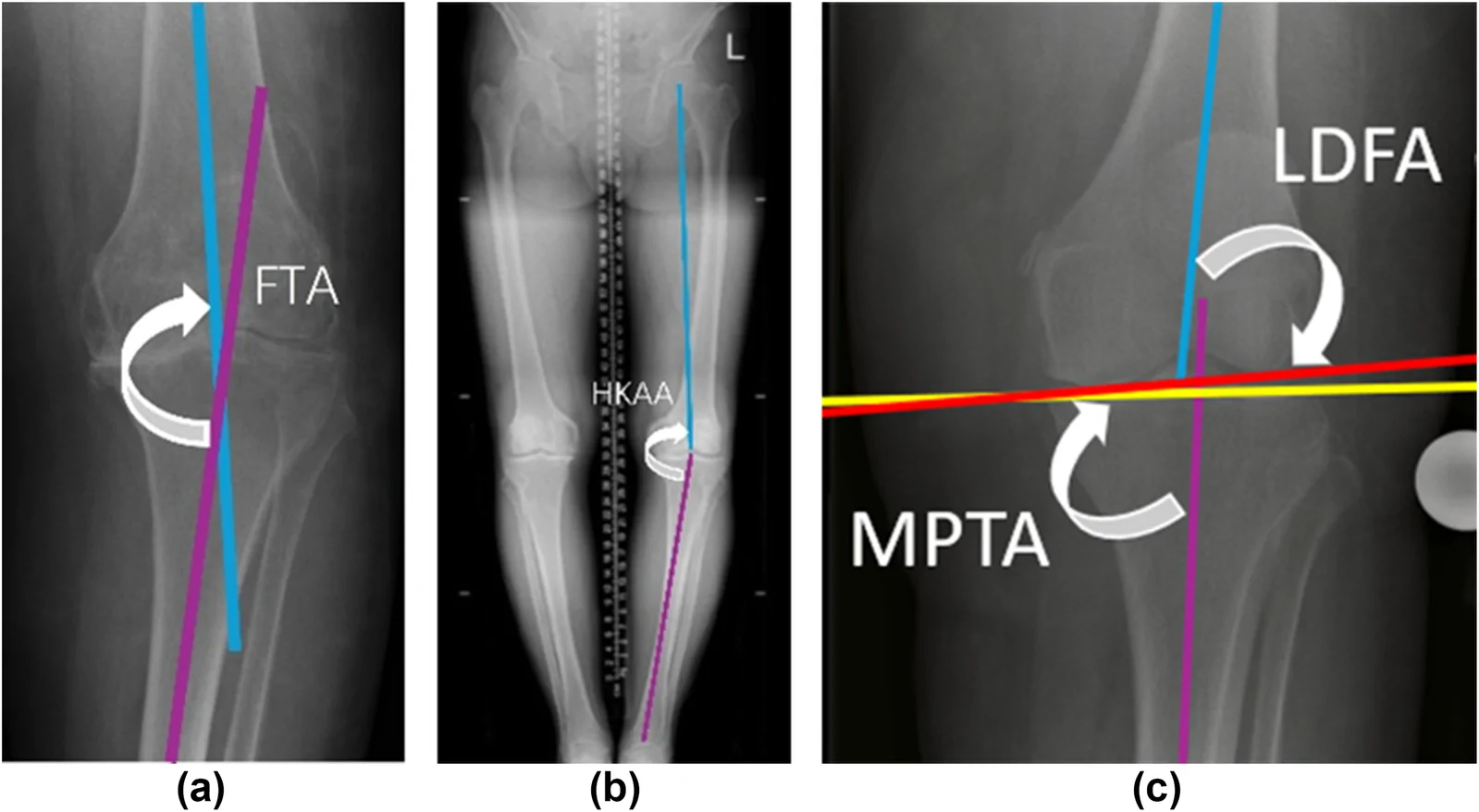
An illustration of four widely used angles (FTA, HKAA, medial proximal tibial angle and LDFA). In practice, they can also be defined using their supplementary angles [11], [12], [13], [33].

Patellofemoral alignment is also clinically relevant in TKR evaluation, although it has been less extensively studied than tibiofemoral alignment. Because the patella is part of the extensor mechanism and articulates with the femoral component after TKR, abnormal patellar tracking, tilt, subluxation, or altered patellar height may contribute to anterior knee pain, reduced range of motion, instability, and poor functional recovery. Preoperative assessment can help identify patellofemoral abnormalities that may influence surgical planning, whereas postoperative assessment can help determine whether patellar maltracking or component-related factors contribute to residual symptoms or unsatisfactory outcomes [37], [38].

Patella height, which refers to the position of the patella relative to the femur and tibia, was identified as another essential radiographic biomarker for TKR outcomes [39]. Patella height can be assessed by measuring the Insall-Salvati index (ISI) [40], the Caton-Deschamps index (CDI) [41], or the Blackburne-Peel index (BPI) [42]. ISI is the ratio of patellar tendon length to patellar length, useful for assessing the patellar position and identifying patellofemoral joint problems. CDI is the ratio of the distance between the anterior corner of the tibial plateau and the most inferior aspect of the patellar articular surface to the patellar articular surface length. It is valuable in assessing patellar instability [43]. BPI is defined as the ratio of the perpendicular distance between the line projected along the tibial plateau and the inferior aspect of the patellar articular surface to the patellar articular surface length. These above-mentioned ratios, measured in lateral knee radiographs (shown in Fig. 2), are key indicators of patellofemoral disorders such as patella alta and patella baja [39], [40], [41], [42].

**Fig. 2:**
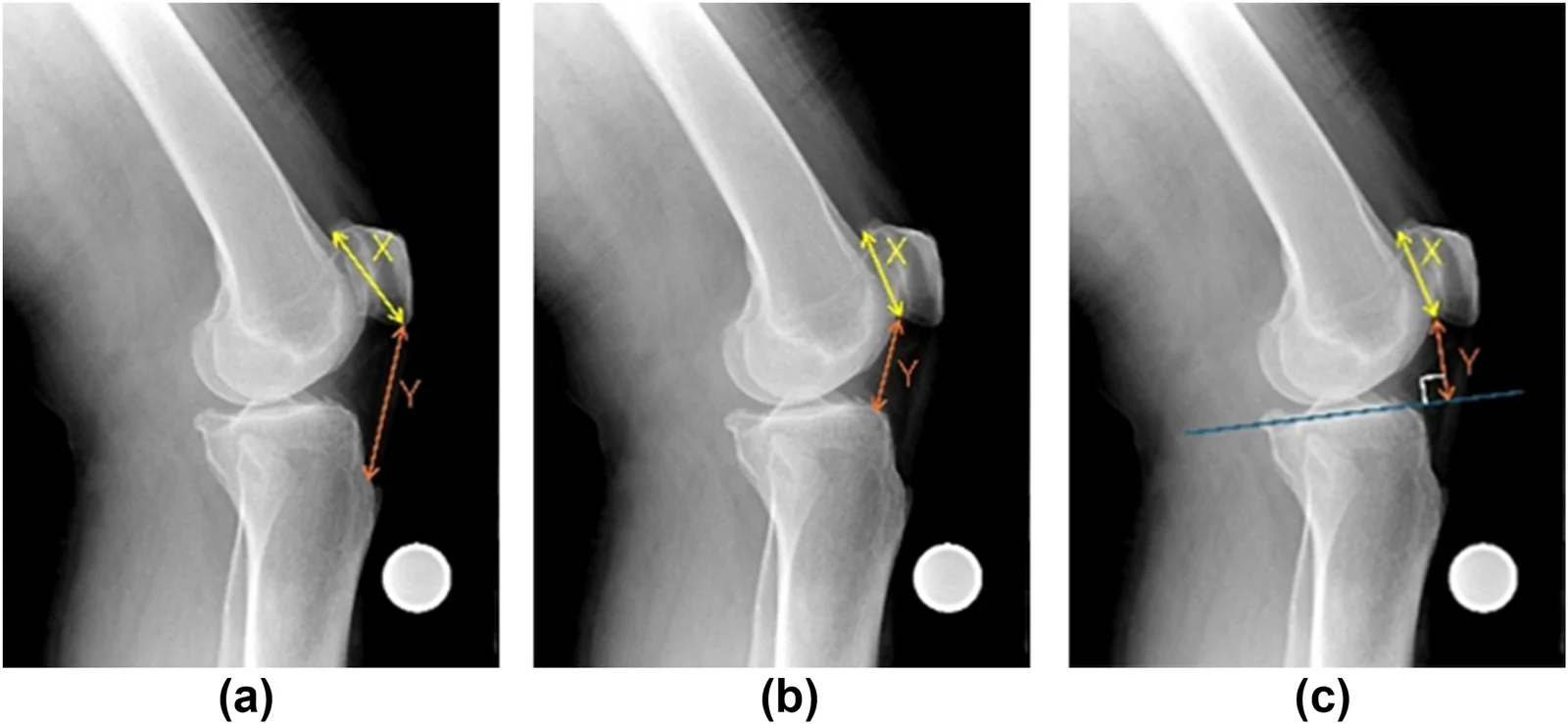
An illustration of the three ratios used for assessing patella height from lateral radiographs. The ISI (a), CDI (b), and BPI (c) are all calculated by Y/X.

## 3 AI-based methods for assessing knee alignment

Machine learning-based methods such as Random Forests (RF) [44] have been widely used in image processing [45], [46], including shape modelling [47], [48], [49], [50], which often provides a basis for knee alignment measurement. Deep learning has shown good performance on many problems in various areas of computer vision [51], [52], [53], [54], [55], [56], [57], [58], [59], [60], [61], [62], [63], [64], [65], [66]. Many popular deep learning models [67] have been used in medical imaging studies, including DenseNet [68], UNet [69], and ResNet [70]. These offer advanced techniques for accurate and efficient processing of radiographs. Specifically, many deep learning-based studies have been developed for automated knee alignment measurements [10], [13], [33].

### 3.1 Landmark detection solutions

Landmark detection [71] is a computer vision technique for identifying points that highlight important features. Two examples illustrating annotated knee joint landmarks are shown in Fig. 3. Landmark detection is often associated with knee alignment as measurements such as angles can be made based on the detected landmark positions. For example, measuring anatomical FTA requires first determining the femoral and tibial anatomical axes, which could be defined by landmarks along the femoral condyles, tibial plateau, and shafts. However, manual annotations are subjective and time-consuming. Automated landmark detection can improve the efficiency and the accuracy of knee alignment measurements, thereby supporting more effective diagnostic and treatment planning for knee joint disorders.

**Fig. 3:**
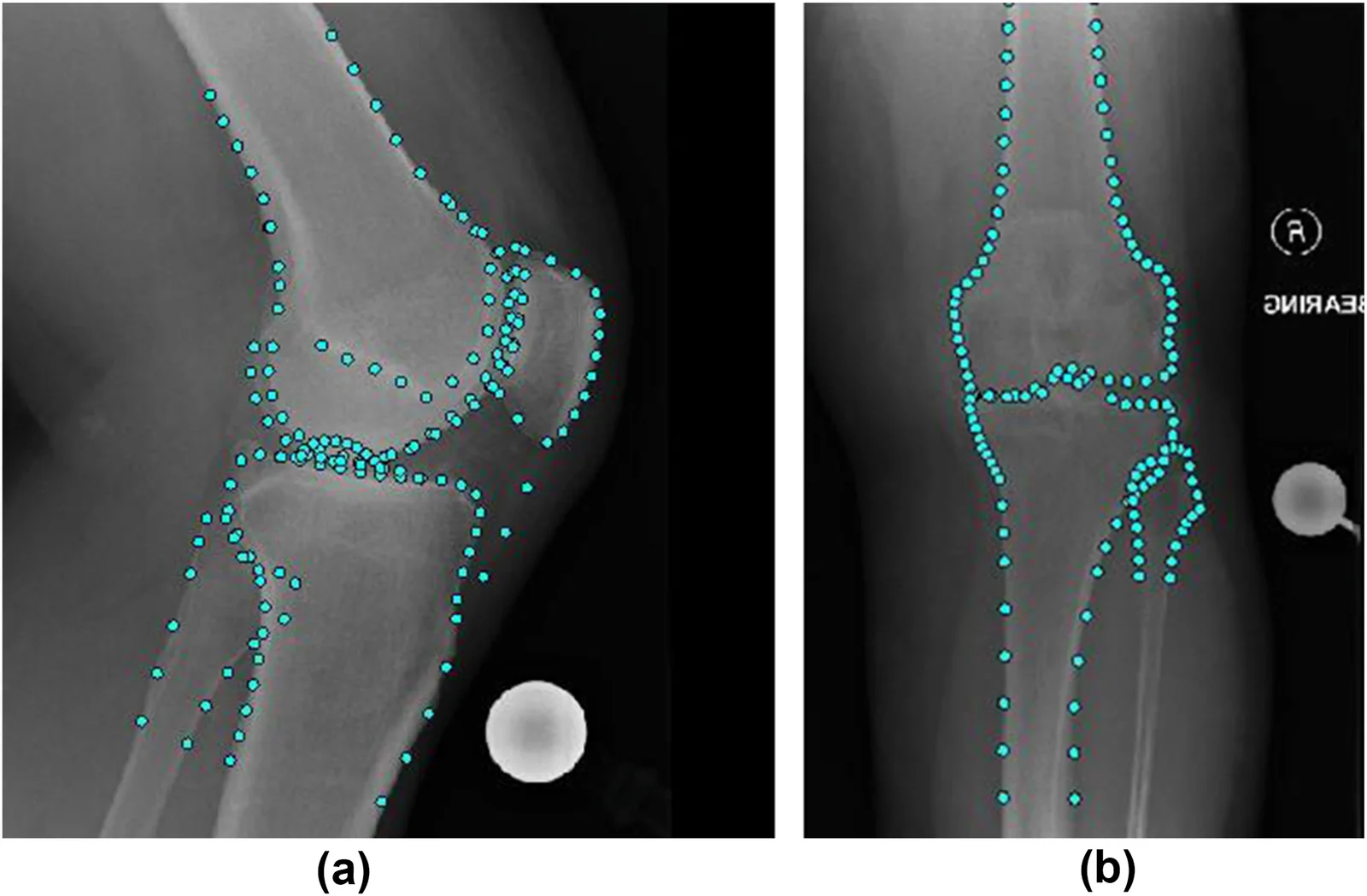
Landmark examples in lateral (a) and AP (b) knee radiographs.

Deformable models can provide effective solutions for automated landmark annotations when combined with model matching techniques [47], [48]. These models are flexible curves or surfaces defined within an image domain, offering the necessary degrees of freedom to adapt the model to diverse shapes. Notable examples include Active Shape Models [72] and Active Appearance Models [73], both based on Statistical Shape Models (SSMs). SSMs capture shape variability across a training dataset, enabling the representation and analysis of population-level shape variations. These models generally require training to learn the shape variability or appearance of the target item. Recently, deformable models have been combined with machine learning [47], [48], [49], [50], [74], [75].

Machine learning methods, particularly RF-based approaches combined with SSMs, have been applied to radiographic landmark detection. For example, Lindner et al. [48] used RF regression voting with constrained local models to localise landmarks in pelvic, knee, and hand radiographs. A few candidate positions were produced by a global search with a detector. Each was then refined using an SSM together with local detectors for each model point. Both global and local refinements relied on RF regression to vote for optimal positions, ensuring robustness and accuracy. In two-fold cross-validation on 500 radiographs, 87 knee contour points were localised, achieving a mean point-to-curve (P2C) error of less than 1 mm in 99% of all cases, demonstrating high accuracy and efficiency.

Deep learning-based methods, particularly neural networks, have also been applied for radiographic landmark detection. Compared with classic landmark regression approaches, heatmap-based landmark regression offers greater robustness. This strategy first generates a probability heatmap for each landmark, and landmark coordinates are subsequently obtained from the heatmap, typically using either the centroid or the maximum response location. Much of the current research has used heatmap-based methods to localise landmarks on pelvis rather than knee radiographs. Davison et al. [76] proposed an automated pelvis landmark detection approach based on heatmap prediction using UNet. They predicted a Gaussian blob for each landmark and estimated displacement of every pixel from each target landmark. Evaluation was performed using relative point-to-point (P2P) and P2C distances, defined as percentile-based errors normalized to the reference length (femoral shaft width). They reported better performance than [48] with a larger and different dataset including radiographs of hips from children aged between 2 and 11 years. Using 1000 images, their method achieved a mean P2C error of 9.0% at the 99th percentile, outperforming the 17.1% error obtained with the method in Ref. [48], though the median P2P error was slightly higher (6.7% vs. 5.9%). Huang et al. [77] propose a landmark detection framework for pelvic X-rays that enhances a base detector by topology-aware constraints and pelvis-focused feature enhancement, ultimately refining 14 anatomical landmark coordinates. The detection head predicts heatmaps, which are post-processed through spatial relationship modelling to enforce anatomical structure consistency. Evaluated on two public and one private dataset of around 850 images, the method achieved a best mean radial error of 0.71 mm and a successful detection rate of ≈97% at 4 mm, outperforming baseline detectors that lack topology-aware refinement. Pei et al. [78] proposed an attention mechanism of combining multi-dimension information based on separating spatial dimension in the pelvis X-ray landmark detection. The attention modules were inserted into the skipped connections of UNet to form a novel structure. Their method outperformed UNet, HR-Net [79], and CE-Net [80], achieving average P2P errors of 3.14 mm versus 3.57 mm, 3.61 mm, and 3.39 mm, respectively. Mulford et al. [81] developed a single CNN model to annotate certain anatomical structures and landmarks on AP pelvis radiographs. A total of 1100 AP pelvis radiographs were manually annotated by 3 reviewers. The model segmented 22 structures, including 7 landmark points, with Euclidean distance errors for landmarks ranging from 1.9 to 5.6 mm. Notably, most of these deep learning-based landmark detection studies focused on pelvic or hip radiographs rather than knee radiographs. Nevertheless, their heatmap-based and segmentation-based strategies could potentially be adapted for knee radiographs to support knee OA assessment and TKR outcome prediction.

Relatively few deep learning-based studies have specifically targeted landmark detection in knee radiographs. Tiulpin et al. [64] used Hourglass-based networks [82] to regress the knee landmark positions from AP knee radiographs more efficiently. The results demonstrated that over 75% of P2P errors were below 2 mm, and over 90% were below 2.5 mm, both of which surpass the performance reported in Ref. [48]. Xiao et al. [83] localised anatomical landmarks in knee radiographs from the OAI dataset by combining heatmap regression with a GCN. By representing landmarks as a graph, the model effectively captures structural information, refining landmark coordinates through a cascade of GCNs. Their model achieved a mean P2P error of 0.84 mm and the successful detection rate of 71.2% at 1 mm. 93.9% of the P2P errors were below 2 mm. Fewer studies focused on other radiographic views, such as lateral or skyline. Tuya et al. [84] detected knee landmarks from skyline radiographs and used these landmarks to further measure patellofemoral joint parameters. Their method achieved 88.9% and 82.2% of errors below 2 mm on the original and hold-out test sets, respectively.

A summary of these landmark detection solutions in lower limb radiographs is shown in Table 3.

**Table 3:** Overview of technical solutions for landmark detection in lower-limb radiographs. (RF: random forest; SSM: statistical shape model; CNN: convolutional neural network; GCN: graphic convolutional network; GT: ground-truth; P2C: point-to-curve; EV: external validation; P2P: point-to-point; MRE: mean radial error; SDR: successful detection rate; PCK: percentage of correct keypoints).

Studies	Diseases Entities (Motivation)	Data (size)	Techniques	Evaluation and performance
[48]	Hip and knee OA	AP pelvic (839) and knee (500) radiographs (arcOGEN Consortium), AP hand (564) radiographs of children.	RF, SSM	**GT:** Manual expert landmark annotations. **Performance:** Mean localization error < **0.7 mm** (95% of images) for the pelvis and knee, < **1.1 mm** (95% of images) for the hand; knee P2C distance < **1 mm** in 99% of images. **EV:** No.
[76]	Paediatric hip disorder	Proprietary dataset of hip radiographs (1696) of children aged 2–11 years.	UNets	**GT:** The average of manual landmark annotations from two trained annotators. **Performance:** Relative mean P2C distance of **9.0%** and relative median P2P distance of **6.7%**, outperforming the previous method (17.1% P2C error). **EV:** No.
[77]	No OA-specific focus	CGMH-PelvisSeg (400), a public pelvic radiograph dataset (150), and one private in-house dataset (300).	Pelvis Extraction module added to CNNs	**GT:** 14 pelvic landmarks manually annotated by a pelvic surgeon and reviewed by a medical imaging practitioner. **Performance:** MRE of **0.71 mm** with SDRs of **85.9%, 90.8%, 96.0%, and 96.7%** at **2, 2.5, 3, and 4 mm**, respectively. **EV:** No.
[78]	Hip OA, developmental dysplasia of the hip, femoroacetabular impingement	OAI open-source pelvic radiograph dataset (524 selected).	U-shaped CNNs, attention	**GT:** Eight pelvic landmarks manually annotated by a radiologist and reviewed by an orthopaedic surgeon. **Performance:** Mean P2P distance of **3.14 mm** with SDRs of **8.65%, 33.89%, 59.25%, and 78.61%** at **1, 2, 3, and 4 mm**, respectively. **EV:** No.
[81]	No OA-specific focus, support total hip arthroplasty workflows	Institutional total hip arthroplasty radiography registry (Mayo Clinic), including pre- and post-operative AP pelvis/hip radiographs (1100).	UNet	**GT:** 22 anatomical structures manually annotated by three trained annotators and reviewed by two orthopaedic surgeons. **Performance:** Mean Dice scores of **0.88** for shape structures and **0.80** for line structures, with landmark localization errors of **1.9**–**5.6 mm**. **EV:** No.
[64]	Knee OA	Training: OAI knee radiographs (748). Testing: Two independent datasets (161, 209) from Oulu University Hospital.	Hourglass networks	**GT:** 16 anatomical landmarks manually annotated. **Performance:** PCKs of **93.5%** (Dataset A) and **92.1%** (Dataset B) at the **2.5 mm** threshold, with < **1% outliers** in both datasets. **EV:** Yes.
[83]	Knee OA	OAI knee X-ray dataset (748).	GCNs	**GT:** Pre-existing manual landmark annotations from the OAI dataset. **Performance:** MRE of **0.84 mm** with SDRs of **71.18%, 87.04%, 93.92%, and 97.11%** at **1, 1.5, 2, and 2.5 mm**, respectively. **EV:** No.
[84]	Patellofemoral OA	Single-center Laurin-view patellofemoral joint radiographs (1431).	UNets	**GT:** Mean of the first measurements from three radiologists. **Performance:** PCK of **88.86%** in the internal test set and **82.19%** in the hold-out test set at the **2 mm** threshold. **EV:** No.

### 3.2 AI-based knee alignment assessment

#### 3.2.1 Angles

Several AI-based approaches have been proposed for automatically measuring knee alignment angles from full-leg radiographs. Moon et al. [85] developed a deep learning-based system to detect lower limb alignment automatically, rapidly, and accurately by using AP standing full-leg radiographic data of lower limbs. The alignment was comprehensively measured by calculating mechanical lateral proximal femoral angle, mechanical LDFA, mechanical MPTA, mechanical lateral distal tibial angle, mechanical axis deviation, joint line convergence angle, mechanical FTA, anatomical medial proximal femoral angle, anatomical LDFA, neck shaft angle, anatomical MPTA, anatomical FTA, and the length of femur, tibia, and the full limb. This algorithm includes region of interest (ROI) detection with You Only Look Once (YOLO)v5 [86], segmentation with HarDNet-MSEG [87], and landmark detection. Using radiographs from 770 patients collected between 2016 and 2020, the system achieved high agreement with radiologist measurements, with intraclass correlation coefficients (ICC) of 0.920–0.995, mean absolute errors (MAE) of 0.46–0.87°, and root mean square errors of 0.54–1.46°. Jo et al. [88] presented a CNN-based anatomical landmark recognition and angle measurement model for femur, tibia, and implant components using full-leg preoperative and postoperative radiographs. 15 anatomical landmarks were marked by two orthopaedic surgeons. Mechanical LDFA, MPTA, JLCA, and HKAA were then measured after extracting the ROIs. The system demonstrated excellent reliability, with inter-observer ICC values greater than 0.98 and MAE of 0.22–0.72°, indicating performance comparable to specialist measurements. Tack et al. [89] proposed a fully automated method for knee alignment quantification using YOLOv4 [90] for ROI detection of the hip, knee, and ankle, followed by ResNet regression of landmark coordinates. Their system produced HKAA measurements highly consistent with expert annotations, with an ICC of 0.98 and external validation. Chen et al. [10] used ResNet to automatically measure HKAA on full-leg radiographs without landmark positions. However, their results showed different levels of agreement depending on the imaging method, with knee radiographs taken using a non-positioning frame demonstrating higher reliability (ICC = 0.90), compared to those taken within a positioning frame (ICC = 0.76).

However, full-leg radiographs are not always undertaken in clinical practice, and standard AP knee radiographs are often the main imaging modality. Thus, automatic angle prediction from standard weight-bearing knee radiographs is clinically valuable. Wang et al. [33] directly predicted anatomical FTA and HKAA from PA knee radiographs without explicitly detecting landmark positions, although HKAA is conventionally measured from full-limb images. In this study, CNNs with densely connected final layers were trained to analyse PA knee radiographs from the OAI database. Separate models were trained for each angle, with mean squared error as the loss function. Heat maps were used to identify the anatomical features within each image that most contributed to the predicted angles. DenseNet achieved high accuracy, with MAE of 0.8° for anatomical FTA and 1.7° for HKAA. Agreement was excellent for anatomical FTA (ICC>0.9 across all CNNs), but lower for HKAA (ICC = 0.7–0.9), reflecting reduced robustness. Cullen et al. [11] predicted anatomical FTA with two different approaches in AP knee radiographs using anatomical landmark positions detected by RFRV-CLM. Strong agreement was found between the automated and clinical measurements of preoperative anatomical FTA (ICC>0.95). The postoperative agreement between the automated and clinical measurements was lower (ICC = 0.7–0.8). Hu et al. [13] applied Hourglass networks to detect anatomical landmark positions in AP knee radiographs and subsequently generate anatomical FTA measurements using the same approaches as [11]. Their system showed higher overall accuracy in landmark detection task compared with RFRV-CLM and stronger agreement between the automated and clinical measurements than RFRV-CLM [11], though the postoperative agreement was also lower than the preoperative one.

#### 3.2.2 Ratios

AI-based approaches have also been developed to measure patellar alignment ratios, particularly from lateral knee radiographs. Ye et al. [91] developed a CNN-based system for automatic patellar height measurement based on detected landmarks in lateral knee radiographs, focusing on ISI and CDI. Tested on 200 left and 200 right knee radiographs, the system achieved performance comparable to manual measurements by three radiologists (ICC>0.9). Kwolek et al. [92] used YOLO and UNet to detect the ROI and measure patella height with CDI and BPI. Good agreement between the orthopaedic surgeons’ measurements and results of their algorithm was achieved (ICC>0.75, standard error for single measurement < 0.014). Liu et al. [93] used ResNet and HR-Net to automatically measure ISI. The system performed excellent in keypoint detection tasks and was highly consistent with the manual measurements of ISI (ICC, 0.809–0.885).

A summary of technical AI-driven solutions for knee alignment assessment is shown in Table 4. The most promising methods were alignment-focused models in OA-specific, OAI, or arthroplasty-relevant cohorts [10], [11], [13], [33], [85], [86]. Landmark-based methods provide interpretable anatomical measurements but require landmark annotation or segmentation [11], [13], [85], [86]. By contrast, landmark-free models that directly estimate HKAA or FTA from radiographs offer a more convenient workflow, avoiding explicit landmark localization and reducing annotation burden [10], [33]. These methods may be especially useful for large-scale OA screening and research, although robust external validation remains essential. Patellar-height-only models were considered less directly relevant to OA alignment evaluation because they mainly assessed patellofemoral morphology and often excluded moderate or severe OA [91], [92], [93].

**Table 4:** Overview of technical AI-driven solutions for knee alignment assessment. (LDFA: lateral distal femoral angle; MPTA: medial proximal tibial angle; LDTA: lateral distal tibial angle; LPFA: lateral proximal femoral angle; MPFA: medial proximal femoral angle; JLCA: joint line convergence angle; FTA: femorotibial angle; HKAA: hip-knee-ankle angle; ISI: Insall–Salvati index; CDI: Caton–Deschamps index; BPI: Blackburne–Peel index; YOLO: you only look once; CNN: convolutional neural network; RFRV-CLM: random forest regression-voting constrained local model; GT: ground-truth; ICC: intraclass correlation coefficient; MAE: mean absolute error; RMSE: root mean square error; EV: external validation; pre-op: pre-operative; post-op: post-operative).

Studies	Diseases Entities (Motivation)	Measurements	Techniques	Data (size)	Landmarks	Evaluation and performance
[85]	No OA-specific focus	LDFA, MPTA, LDTA, LPFA, MPFA, JLCA, FTA	YOLOv5, HarDNET-MSEG	Single-center AP standing full-leg radiographs (450).	Required	**GT:** Radiologist measurements. **Performance:** ICCs were **0.920**–**0.995**, with MAEs of **0.46**–**0.87°** and RMSEs of **0.54**–**1.46°**. **EV:** No.
[88]	Knee OA	LDFA, MPTA, JLCA, HKAA	CNNs	Single-center full-leg standing radiographs (11,212).	Required	**GT:** Orthopaedic specialist annotations/measurements. **Performance:** Inter-observer ICCs ≥ **0.98** for all four angles; MAEs **0.22**–**0.72°**. **EV:** No.
[89]	Knee OA	HKAA	YOLOv4, ResNet	OAI full-leg radiographs (3843).	Required	**GT:** Manual landmarks and expert OAI angle readings. **Performance:** HKAA error was **0.09**±**0.73°** versus Cooke and **0.18**±**0.67°** versus Duryea; ICC was **0.97**–**0.98**. **EV:** Yes.
[10]	Knee OA	HKAA	ResNet	AP/PA full-leg radiographs and knee radiographs from OAI plus Austrian/US sites (8878).	Not required	**GT:** Automated HKAA from full-leg radiographs. **Performance:** On knee radiographs, ICC was **0.76** for SynaFlexer™ images, and **0.90** for non-positioning frame images, and MAEs were **1.56°** and **2.10°**; cropped full-leg images achieved ICC of **0.94** and MAE of **0.98°**. **EV:** No.
[33]	Knee OA	Anatomical FTA, HKAA	DenseNet, Inception-ResNet v2	OAI database; PA knee radiographs (6149 for FTA, 2351 for HKAA).	Not required	**GT:** OAI-reported FTA and HKAA. **Performance (DenseNet):** FTA—MAE **0.82°**, ICC **0.91**, **98.7%** within 3°; HKAA—MAE **1.73°**, ICC **0.80**, **83.9%** within 3°. **EV:** No.
[11]	Knee OA	Anatomical FTA	RFRV-CLM	Single-center AP weight-bearing knee radiographs (1775).	Required	**GT:** Orthopaedic surgeon measurements. **Performance:** Automatic vs manual measurements achieved **ICC 0.98/0.96** and MAEs of **0.8°/0.7°** (pre-/post-op); automatic vs clinical measurements achieved **ICC 0.97** (pre-op) and **0.78** (post-op). **EV:** No.
[13]	Knee OA	Anatomical FTA	Hourglass network	Standard anteroposterior knee radiographs (1775).	Required	**GT:** Orthopaedic surgeon measurements. **Performance:** Automatic vs manual measurements achieved **ICC 0.98/0.96** and MAEs of **0.8°/0.7°** (pre-/post-op); automatic vs clinical measurements achieved **ICC 0.97** (pre-op) and **0.78** (post-op). **EV:** No.
[91]	No OA-specific focus	ISI, CDI	VGG16	Single-center lateral knee radiographs (1418).	Required	**GT:** Orthopaedic surgeon measurements. **Performance:** Clinical agreement for anatomical tibiofemoral angle was **ICC 0.97** pre-operatively and **0.86** post-operatively. **EV:** No.
[92]	No OA-specific focus	CDI, BPI	UNet, YOLO	Single-center high-resolution lateral knee radiographs (184 for UNet, 900 for YOLO).	Required	**GT:** Mean of manual landmark annotations from three radiologists. **Performance:** Predicted the ISI, CDI, and KI with **ICC 0.91**–**0.96**, **MAE 0.02**–**0.06**, and **RMSE 0.02**–**0.10**; performance for the modified CDI was lower (**ICC 0.62**–**0.65**). **EV:** No.
[93]	No OA-specific focus	ISI	ResNet, HR-Net	Multicenter lateral knee radiographs (2341).	Required	**GT:** Orthopaedic surgeon measurements. **Performance:** Automatic measurements showed excellent agreement with surgeons for CDI and BPI (**ICC 0.79**–**0.88**). **EV:** No.

### 3.3 Commercial products for automated knee alignment assessment

Several commercial artificial intelligence (AI) solutions provide automated knee alignment assessment, including picture archiving and communication systems (PACS)-integrated tools, but typically rely on full-leg radiographs for measurements such as HKAA. ImageBiopsy Lab^1^ has developed an AI-powered software solution to automate and standardise musculoskeletal imaging diagnostics. Their software automatically measures JLCA, FTA, and HKAA, achieving near-perfect agreement with reference standards (ICC close to 1; MAEs ≤1°) [94], [95]. Moreover, the intra-observer ICC for automated approach is better than manual ones [96], demonstrating more reliable measurements. Gleamer^2^ has developed AI-driven software like BoneMetrics, which automates measurements including knee alignment on full-leg standing radiographs, delivering accurate and reproducible HKAA values with excellent agreement compared to ground truth [97]. TechCare Bones developed by Milvue^3^ also provides an automated solution to assessing knee alignment including HKAA from full-leg radiographs, but no literature is available to provide evidence of its performance.

For the analysis of standard weight-bearing knee radiographs, current commercial products are typically limited to grading knee osteoarthritis. Although full-leg radiographs provide more comprehensive anatomical information, it is clinically valuable to explore the feasibility of alignment measurement using standard weight-bearing knee radiographs.

Currently available commercial products for radiographic knee alignment measurement are summarised in Table 5.

**Table 5:** Available commercial products for radiographic knee alignment assessment. (HKAA: hip-knee-ankle angle; FTA: femorotibial angle; JLCA: joint line convergence angle; LDFA: lateral distal femoral angle; MPTA: medial proximal tibial angle; LDTA: lateral distal tibial angle; LPFA: lateral proximal femoral angle; ICC: intraclass correlation coefficient; MAE: mean absolute error; RMSE: root mean square error; MSE: mean square error).

Companies	Measurements	References	Modalities	Evaluation (performance)
ImageBiopsy Lab	HKAA, FTA, JLCA, LDFA, MPTA, LDTA, LPFA	[94], [95], [96]	Full-leg X-ray	ICC (>0.9); MAE (<1°)
Gleamer	HKAA	[97]	Full-leg X-ray	ICC (>0.99); RMSE (0.37); MSE (0.30)
Milvue	HKAA	N/A	Full-leg X-ray	N/A

## 4 Discussions and conclusions

Knee alignment is associated with TKR outcomes: varus and valgus deformities may predict greater postoperative improvement [27] but also increased revision risk [9], while postoperative malalignment is associated with long-term component failure [31]. However, no clear relationship was found between knee alignment and postoperative outcomes when assessed using OKS [32], warranting evaluation using additional outcome measures.

Tibiofemoral alignment is typically measured using angles such as FTA, MPTA, LDFA, and HKAA, while patella height is evaluated through ratios including ISI, CDI, and BPI. Landmark detection identifies key points in radiographs, enabling subsequent knee alignment measurements. Both machine learning and deep learning-based approaches, such as RFRV-CLM [47], [48], [49], [50] and CNNs [64], have been developed for this purpose. CNNs may offer greater reliability and robustness in landmark detection but are not yet more accurate than RFRV-CLM. A more accurate pipeline may involve RFRV-CLM to refine the point positions initialised by CNNs.

AI-based techniques could provide accurate and efficient knee alignment assessments. Many approaches showed excellent accuracy comparable to manual measurements [10], [11], [13], [85], [88]. Some angles, such as HKAA, traditionally require full-leg radiographs. However, acquiring and pre-processing these images can be time-consuming, and they result in increased radiation exposure. Therefore, predicting these measurements from standard weight-bearing knee radiographs would be clinically valuable. Current HKAA prediction approaches from standard weight-bearing knee radiographs remain unstable, highlighting the need for more robust and reliable models [33]. Most existing alignment assessments methods rely on landmark detection, though direct angle prediction could provide an alternative pathway [10], [33]. Furthermore, many studies lack radiologist-provided manual annotations or independent validation, limiting confidence in generalisability [10], [33]. Future work should emphasise external validation on unseen data to demonstrate clinical applicability and reliable performance across practice settings.

Most current methods for knee alignment assessment focus on the AP and lateral views. Unlike 3D modalities such as MRI, combining different X-ray views is more challenging, and most existing approaches for landmark detection and alignment measurement only analyse a single view. Developing methods to combine multiple radiographic views could provide more comprehensive information [98].

While the link between knee alignment and TKR outcomes is well established, no automated approaches currently predict TKR outcomes directly from radiographic alignment. Developing end-to-end systems that either integrate automated alignment measurement with outcome prediction, or directly infer outcomes from radiographs, represents a promising research direction.

In conclusion, radiographic knee alignment biomarkers provide clinically meaningful information for TKR planning, evaluation, and outcome prediction, but their interpretation should be integrated with PROMs and other patient-specific factors. AI-based approaches can automate alignment assessment and reduce the burden of manual measurement, but further work is required before they can be widely adopted in clinical practice. Future studies should focus on externally validated, interpretable, and clinically integrated models that can measure both tibiofemoral and patellofemoral alignment from routinely acquired radiographs and link these imaging biomarkers directly to preoperative risk stratification, postoperative assessment, and long-term TKR outcomes.

## Contributions

The authors have made substantial contributions to the following sections.

• Conception and design (ZH, AT, TFC, CL).
• Drafting of the article (ZH).
• Revising the article critically for important intellectual content (ZH, DC, DSJ, AT, TFC, CL).
• Final approval of the article (ZH, DC, DSJ, AT, TFC, CL).

## Generative AI

Generative AI and AI-assisted technologies were NOT used in the preparation of this work.

## Funding source

ZH is funded by European Laboratory for Learning and Intelligent Systems Unit Manchester. CL is funded by a Sir Henry Dale Fellowship jointly funded by the Wellcome Trust and the 10.13039/501100000288Royal Society (223267/Z/21/Z), DC was supported by the same award. This research was funded in whole, or in part, by the 10.13039/100010269Wellcome Trust [Grant number 223267/Z/21/Z]. For the purpose of open access, the authors have applied a CC BY public copyright licence to any Author Accepted Manuscript version arising from this submission.

## Conflict of interest

The authors have no competing interests to declare.

## Footnotes

1 https://www.imagebiopsy.com/.

2 https://www.gleamer.ai/.

3 https://www.milvue.com/en/solutions/techcarebones/.
